# Development and pilot evaluation of a home-based palliative care training and support package for young children in southern Africa

**DOI:** 10.1186/s12904-016-0114-7

**Published:** 2016-04-09

**Authors:** Sara Naomi Naicker, Linda Richter, Alan Stein, Laura Campbell, Joan Marston

**Affiliations:** Human Sciences Research Council, Human & Social Development Programme, 5th Floor, The Atrium, 430 Peter Mokaba Ridge, Berea, Durban, South Africa; WITS/MRC Developmental Pathways for Health Research Unit, Department of Paediatrics, Faculty of Health Sciences, University of Witwatersrand, Johannesburg, South Africa; DST-NRF Centre of Excellence in Human Development, University of the Witwatersrand, 1 York Road, Parktown, Johannesburg, South Africa; Human Sciences Research Council, 5th Floor, The Atrium 430 Peter Mokaba Ridge, Berea, Durban, South Africa; Department of Psychiatry, University of Oxford, Oxford, OX3 7JX UK; Wits/MRC Rural Public Health and Health Transitions Research Unit [Agincourt], School of Public Health, Faculty of Health Sciences, University of the Witwatersrand, Johannesburg, South Africa; School of Clinical Medicine, University of KwaZulu-Natal, Durban, South Africa; International Children’s Palliative Care Network, Assagay, KwaZulu-Natal, South Africa

**Keywords:** Southern Africa, Paediatric, Palliative care, Home-and community-based, Caregiver, Young children, HIV

## Abstract

**Background:**

The leading cause of death among young children in southern Africa is complications due to HIV infection and, in South Africa, over a third of all deaths of children younger than five are associated with HIV infection. There is a great and urgent need for children’s palliative care in Africa, whether HIV-related or not. It is often not possible for sick children and their carers to attend clinics and hospitals cannot accommodate children for long periods of time. As a result children are often cared for in their own homes where caregivers require support to provide informed and sensitive care to reduce children’s suffering. Home-care places a heavy burden on families, communities and home- and community-based care workers.

**Methods:**

This project involved the development and pilot evaluation of a training and support package to guide home and community-based care workers to help caregivers of seriously ill young children at home in southern Africa. A number of research methods were used, including a cross-sectional survey of content experts using the Delphi technique, participatory action research with photo elicitation and qualitative thematic analysis.

**Results:**

Because the palliative care needs of these children are complex, the package focuses on delivering 9 key messages essential to improving the quality of care provided for young children. Once the key messages were developed, culturally relevant stories were constructed to enhance the understanding, retention and enactment of the messages. The various research methods used, including literature reviews, the Delphi technique and photo-elicitation ensured that the content included in the package was medically sound and culturally relevant, acceptable, feasible, and comprehensive. The end product is a home-based paediatric palliative care training and support package in English designed to help train community workers who are in a position to support families to care for very sick young children at home as well as to support families in looking after a very sick child.

**Conclusion:**

A pilot study to assess the training and support package found it to be useful in delivering the key messages to caregivers. The training component was found to be feasible. It is concluded that the package offers a practical means of integrating palliative care with home-based care. Further implementation and evaluation is needed to establish its utility and impact.

**Electronic supplementary material:**

The online version of this article (doi:10.1186/s12904-016-0114-7) contains supplementary material, which is available to authorized users.

## Background

### Overview of the need for home-based paediatric palliative care for young children in southern Africa

The humane and effective care of children with life-limiting and life-threatening disease in southern Africa is an important public health challenge [1]. In Africa, children are more likely to face illness and death before the age of 5 years than anywhere else in the world [2]. Paediatric wards in South African government hospitals are occupied predominantly by children with HIV- and AIDS-related illnesses [3]. Globally, estimated at 34 % in 2012, HIV treatment coverage for children remains about half that of coverage for adults [4], and many children will return to hospitals as a result of this persistent poor access to consistent antiretroviral therapy and their subsequent deteriorating health [5], facing stressful and demoralizing cycles of repeated hospital admissions. Not all sick children reach a healthcare facility; long distances, limited transport and poverty restrict access to hospitals where paediatric HIV care is provided [6]. Of those who are admitted to a hospital, many are discharged when no further advantage of hospital care is seen, and sick children are then cared for in their own homes. However, this places a heavy burden on families, communities and home- and community-based health and social care workers to ensure sensitive and competent care to minimise suffering and maximise the quality of life of young children and their families. The aim of home-based care is to broaden healthcare coverage and reach the most vulnerable families. It rests on the conviction that families are well placed, with support from home- and community-based care (HCBC) workers, to play a key role in delivering a continuum of holistic care from onset through illness, chronicity and, potentially, death [7]. The sub-specialty of paediatric palliative care focuses on this holistic, ongoing care for very ill, dying and bereaved children and their families in order to achieve the best quality of life for children living with life-threatening illnesses. Many existing home- and community-based interventions cover components of children’s palliative care; few, however, propose a comprehensive framework that considers the holistic needs of the child and the family. Findings from a recent systematic review of care models and interventions for paediatric palliative care in sub-Saharan Africa found only two studies reporting specifically on paediatric palliative care services [8]. Some home-based programmes also lack support and supervision by professionally trained palliative care providers [9], and only a small number of HCBC workers have been trained in both home-based care and children’s palliative care in South Africa.

### Contextualizing home care for sick young children

In many countries, healthcare facilities are overstretched due to the large number of children presenting with HIV- and AIDS-related illnesses, in addition to other health problems. Consequently, a huge burden is placed on nurses and health care workers. Richter and colleagues have examined the hospital care needs of young children in the context of HIV in KwaZulu-Natal, South Africa [3]. Using video recordings, the routine health care practices, caregiver visits, and challenges to the care of hospitalised children with HIV and AIDS were documented. Many of the children experienced separation anxiety as caregivers were not always able to stay with them. Hospital policies decreased parental contact and sometimes disempowered parents from being involved in the care of their children. In addition, health professionals experienced high levels of burnout as a result of stress. The South African government has adopted and advocated for a home-based care strategy in an attempt to broaden healthcare coverage. The family unit has the primary responsibility of delivering services to the child as well as the greatest capacity to positively influence child health and wellbeing, both prior and subsequent to professional healthcare intervention [10]. Children’s palliative care in sub-Saharan Africa is delivered by a range of healthcare providers, professional and non-professional, and can be situated in healthcare facilities as well as in the child’s home.

Caring for children with HIV infection and other serious illnesses at home has a range of benefits. In many cases home-care is preferable to both the family and the child and requires fewer health service resources [11]. Being cared for at home also facilitates certain cultural practices and removes the constraints placed on family members by restrictive hospital visiting hours [12], allowing greater family involvement particularly during end-of-life care [13]. However, the needs of sick young children, including those with HIV infection, are complex. The model of home-based care works well only when there is a home that is materially appropriate, where an individual or individuals are able to consistently care for the child, and an HCBC worker who can support the provision of “*lay palliative care”* is available [14].

A review of paediatric palliative care for youth with HIV or AIDS identified 21 papers, focusing mainly on physical aspects of care. The authors recommended that future studies focus on broadening the evaluation of paediatric palliative care by examining psychological, social and cultural aspects of care as well as the needs of all stakeholders [15]. A more recent review on paediatric palliative care models, interventions and outcomes in sub-Saharan Africa found only five service descriptions (only four of which were accompanied by evaluation data) which could be categorised as acute, hospice and network care models [8]. In this paper, we describe the development of a palliative care package for the home-care of young children, most of whose lives are limited by untreated HIV or delayed HIV treatment, which can be tailored to suit various service delivery mechanisms (see Additional file 1 for excerpt of package). The aim was to develop a comprehensive package through research activity that sought to understand the context in which young children and their families and communities are facing life-limiting conditions and diseases with little resources [8].

## Methods

The aim of the project was to develop and conduct an initial evaluation of a comprehensive training and support package to assist HCBC workers in supporting the provision of palliative care for very sick young children at home in the southern African context. The study was carried out in three rural areas in KwaZulu-Natal, near Amanzimtoti, Port Shepstone and Pietermaritzburg in South Africa. A number of organizations providing HCBC in this greater KwaZulu-Natal area were approached to participate in the study. The requirement for enrolment was that the organizations provided home-based services in these rural areas where a training and support package would be most needed. Seven HCBC organizations met this selection criterion. Each organization’s involvement in the study varied depending on the feasibility, willingness to participate in a specific component of the study, and the needs of that component of the study. Since it was crucial that the training and support model was suitable to a range of settings, the researchers selected four HCBC organizations working in different rural areas of KwaZulu-Natal and with large enough numbers of HCBC workers to participate in the model development stage. HCBC workers from only two organizations participated in testing the feasibility of the use of stories to relay key messages. All HCBC workers were first language Zulu speakers but were fluent in both spoken and written English. Many, if not all, of the HCBC organizations relied heavily on outside funding and operated with limited resources and overstretched staff. It was important for the researchers not to put any additional burden on the organizations or the HCBC workers. This played a role in how many and which organizations were selected to participate in each component.

**Fig. 1:**
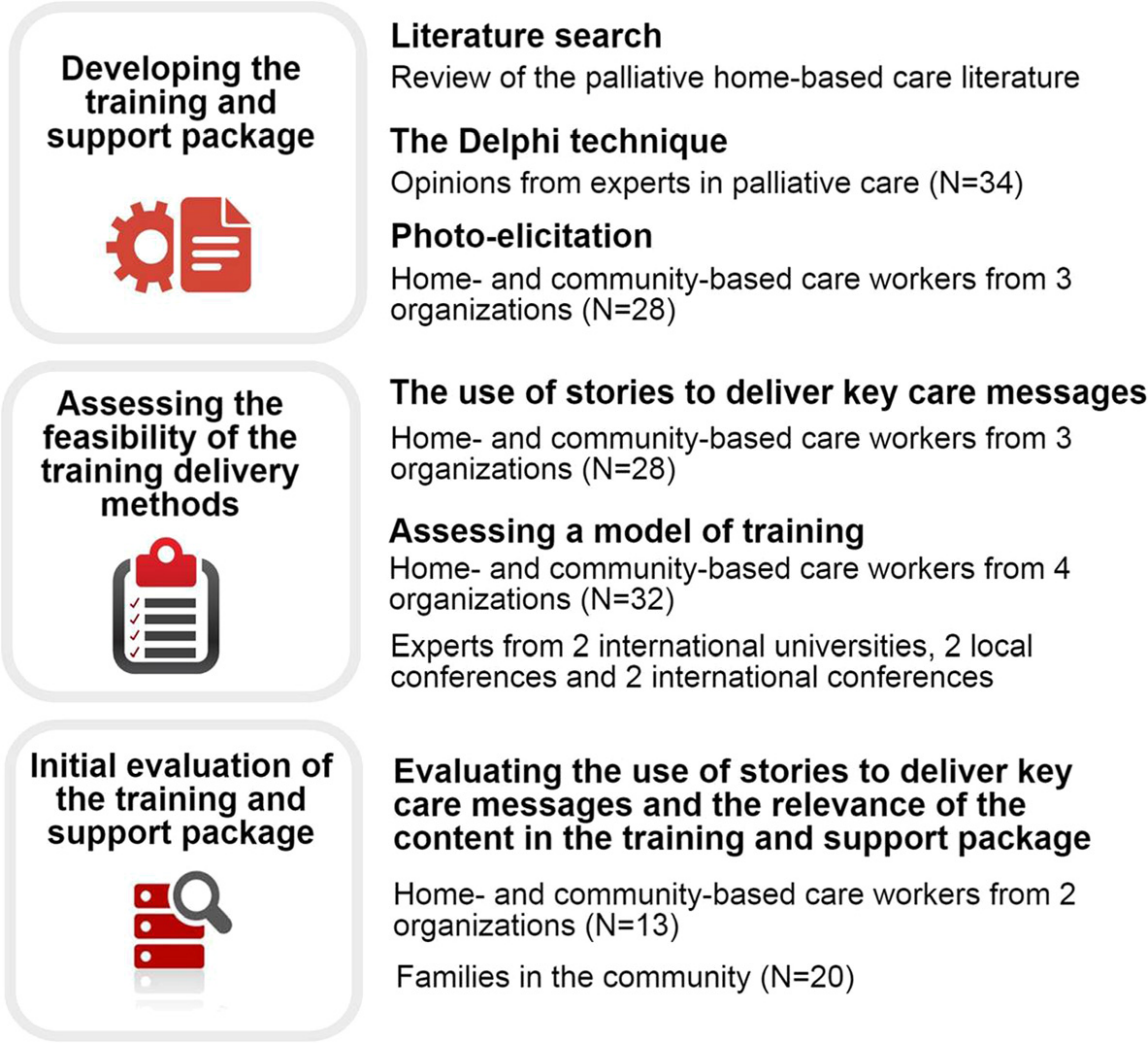
Stages and participation across the study

A three-stage approach (see Fig. 1) was used to undertake the project [16]. In the development stage, a number of methods were used to explore the content to be included in the training and support package. The feasibility stage involved assessing whether stories could be used to convey key messages to address challenges in training HCBC workers in children’s home-based palliative care. We also assessed HCBC workers’ opinions on the content, structure and delivery of a prototypical model of the training and support package. In the evaluation stage an independent assessor examined the retention and perceived usefulness of key messages by both the HCBC workers and the caregivers (referring to the primary caregiver of the child, which may be a parent, grandparent, other relative or any other primary caretaker of a child) they supported. The training component of the package includes material on how to train trainers and how to subsequently train HCBC workers. The support component of the package provides guidance to HCBC workers on how to care for themselves as well as for the families they serve. HCBC workers are trained on how to empower primary caregivers to better care for their own children.

### Developing the training materials

#### Literature search: Identifying and synthesizing relevant studies

Through extensive searches we examined the literature pertaining to the palliative care needs of children, caregivers, families and HCBC workers, focusing on the expectations of home-based care programme managers and those of caregivers, as well as the experiences of HCBC workers. We specifically wanted to focus on the sub-Saharan African context. Both published and unpublished literature was searched since this area of study is still emerging. Five electronic meta-databases namely, Biomed Central, Ebscohost, Pubmed, PsycINFO and Science Direct, were searched for material from 1970 to June 2011. Combinations of specific search terms were used; these included *home-based care, home-based palliative care, home-based paediatric care, children’s palliative care, home-based care worker, community caregiver, community healthcare worker, informal caregiver, child healthcare worker, roles of home-based care workers, practices of home-based care workers, experiences of home-based care workers, Africa.* We also obtained training materials specific to children’s home-based palliative care in Africa from various organizations working in the field, such as the African Palliative Care Association, Hospice and Palliative Care Association South Africa. Papers focusing on the role of HCBC workers were acquired from the South African National Department of Health [17] and other sources [18–22]. Two researchers reviewed the material gathered and synthesized the information in terms of core needs related to home-based palliative care to be dealt with in the training and support package, specifically the expectations of home-based care programme managers, HCBC workers and caregivers, and the experiences of HCBC workers. The majority of the papers focused on the home-based care of adults with HIV and AIDS, though highlighting the need for work on children in this context. There was also a dearth of literature on education and training in the field of paediatric palliative care, a finding consistent with a systematic review of the paediatric palliative care for youth with HIV and AIDS literature [15].

Salient points surfaced from these efforts. Firstly, HCBC workers tend not to have a standardized role, scope of practice or training experience. As a result, the range of duties they are expected to carry out is broad and open-ended. Home-based care programme managers expect HCBC workers, who care for both children and adults, to provide targeted education around HIV prevention, maintain records of visits, administer pain management and assist with the development of care plans that include but are not limited to physical and psychological needs, diet, exercise and personal habits, recreation and home layout [20]. Secondly, there is a discrepancy between what managers and caregivers expect from HCBC workers. Managers tend to focus on practical aspects of care while caregivers also expect their emotional and spiritual needs to be met [17, 18]. Finally, HCBC workers frequently report burnout and distress while carrying out their duties. There are varying levels of support available for HCBC workers in this regard but ample room for improvement [19–22].

The results from the literature review (Table 1) enabled the researchers to prioritize and organise the broad issues that needed to be addressed by a training and support package that was to be comprehensive and realistic to the context under which HCBC workers in sub-Saharan Africa serve the community.

**Table 1:** Summary of expectations placed on HCBC workers from the perspectives of managers and caregivers as derived from literature review

Expectations from programme managers	Expectations from caregivers
• Provide targeted education around HIV prevention • Maintain records of visits • Management of pain • Development and delivery of care plans • Monitor growth and immunization status through activities such as the “Road to Health Card' • Have working knowledge of medicines for children	• Provide physical care – bathing, dressing, eating, using the toilet • Provide general nursing care – pain management, treating wounds, encourage adherence to medication • Assist with household chores – cooking, cleaning, running errands, child care • Provide information and support on disclosure and medication • Transporting patients to health services • Provide advice on family care, and financial and legal aid

#### Soliciting expert views: The Delphi technique

The Delphi technique has been used successfully to identify and organise the opinions of key role players in a particular research field [23]. Electronic technologies are a valuable medium for collecting views from and generating consensus among geographically separated groups of experts. The technique entails formulating a questionnaire to elicit expert opinion on a topic. Existing training material on African home-based paediatric palliative care was reviewed [17, 24–29] and topics related to the provision of care were collated from this literature. From this, a 29-item close-ended questionnaire was developed as well as an open-ended section for additional comments. Themes were developed around topics or areas of the provision of care, and included physical and psychosocial care (including nutrition, danger signs, and emergencies); disclosure of HIV status to the child; communication and play therapy; care for carers; clarifying roles and responsibilities of HCBC workers; training methods and adult learning techniques; and support groups. Respondents were asked to consider whether each item should be included in a training and support package and to rate them according to ‘not necessary’, ‘nice to know but not essential’, and ‘essential’. The questionnaires were posted via group email hosted by the International Children’s Palliative Care network and aimed at providers of paediatric palliative care. Within two weeks of posting, we received the opinions of 34 experts in palliative care from the United Kingdom (20), the United States of America (6), South Africa (5), Canada (1), Tanzania (1) and Zambia (1). The respondents included palliative care nurses (10), team leaders in palliative care (3), regional officer in home-based and palliative care (1), pain and palliative care coordinators (2), palliative care educators (5), public health researchers (7), paediatricians (2), social workers (3) and one medical doctor. Results were organized according to the respondents’ ratings of the level of importance of each item in the context of paediatric palliative care training in Africa. The results of the exercise are reflected in Table 2. Overall, respondents were satisfied with the proposed topics. One area that was emphasized more than any other was the operational needs of a home-based care programme such as monitoring, evaluation and record keeping. Respondents felt that it was important for the content and delivery method of training material to be sensitive to the local situation and for contentious issues such as disclosure of HIV status and the use of traditional medicines to be addressed, although there was no consensus on the issue of discussing HIV infection status/death with children and cultural beliefs played a big role in how this was approached, if at all. It is common in sub-Saharan Africa for caregivers to not disclose a positive HIV test result to a child, even when the child asks questions about their illness [30]. Common reasons for non-disclosure include lack of emotional preparation by the caregiver, lack of knowledge and skills, and a sense of unease in discussing HIV and illness with children. A point made by one respondent was that home-based care workers should be trained to assess when caregivers believe it is appropriate to talk to about HIV infection, dying and death with a young child living with HIV and should be able to facilitate this dialogue.

**Table 2:** Prioritization of aggregated themes by experts through the Delhi technique

Aggregated themes	Aggregated score	Maximum possible score	% of maximum score
Record keeping, monitoring and evaluation	106	108	98
Recognizing and managing child abuse and child neglect	104	108	96
How to train/adult learning methods/carrying out a needs assessment	206	216	92
Care for the carers/recognizing and dealing with burnout	100	108	92
Communication with children (including play)	98	108	90
Physical symptoms assessment and treatment (including nutrition)	286	324	88
How to deal with ethical/moral dilemmas	93	108	86
Clarifying the roles and responsibilities of home-based care workers	93	108	86
Recognizing and dealing with abnormal bereavement (including memory work)/recognizing imminent death	281	324	86
How to run a support group	91	108	84
Recognizing and dealing with emergencies (including danger signs)	174	216	81
Prevention of illness	163	216	75
Psychosocial issues (welfare grant/birth certificate/death certificate/information on burial)	222	324	68
Promoting the role of the family/men	145	216	67
Talking to a child about death/disclosing HIV status to the child	66	108	62
Discourage patients from seeking alternative treatments (traditional medicines/traditional healers)	115	216	53
Community mobilization/ownership of home-based care project/income generation	111	216	51
Training on antiretroviral therapy (ART)	53	108	49

Note: a number of items are included under each theme and are rated along a Likert scale. Items scores are aggregated for each theme and reflected above as the *aggregated score*, dependent on the number of items under a theme a *maximum possible score* is determined, the per cent of the maximum score reflects the aggregated score’s level of agreement and prioritization

Half of the respondents felt that patients should be discouraged from seeking assistance from traditional healers; there was a general consensus that a training and support package for HCBC workers include guidance on how to assess caregivers’ attitudes towards and practices of traditional remedies, and what advice to give. The authors recognise that only 7 of the 34 responses were from Africa, and that future research should employ focus group discussions, postal questionnaires or telephonic interviews in efforts to reach African experts in children’s palliative care.

Topics that were not included in the questionnaire but were suggested by respondents were incorporated into the researchers’ working list and statements were refined in an iterative process. After an acceptable level of consensus, where at least 90 % of the participants in the process agreed on the prioritization of topics, the process was terminated and the material used as a concordant view to be incorporated into the training and support package. Findings from this activity reinforced some of the findings from the literature review. For example, comments received from responses to the questionnaire emphasized the need for their work to be supported by healthcare professionals and cautioned HCBC workers to be aware of the limitations to what they can provide, particularly in contexts of poverty where families may express the need for money and/or food [20]. However, the review pointed out that HCBC workers were mindful of this but did not always have access to professional support and their roles, responsibilities and scope of practice was not always made explicit.

#### Using photo-elicitation to explore HCBC workers experiences and views

Photo-elicitation, or photo-voice, is a community-based participatory action research method [31]. Participants are invited to take photographs that, in their opinion, highlight features of a particular issue or capture their feelings and experiences. The photographs are then discussed in a group setting with the aim of understanding the needs of HCBC workers, and their views and experiences of caring for young children living with HIV at home This is a novel approach in the home-based palliative care setting and in addition to their experiences caring for sick young children, we explored the HCBC workers’ views on the method used. Radley and Taylor [32] demonstrate the suitability and value of this approach in their study aimed at understanding the impact of the physical setting of hospital wards on patient recovery. Among other benefits, photo-elicitation facilitates the development of rapport between participant and researcher, reduces uneasiness in having to answer direct questions as opposed to generating discussion around photographs, acts as a trigger for memory [33] and, when participants themselves have taken the photographs, they represent direct entry points into participant perspectives.

Disposable cameras were given to 28 HCBC workers from three organizations who were asked to take photographs that highlighted features of their experiences of caring for sick, young children. The majority of the participants were female (N = 25) with an average age of 37 years (range 22-60). Seventeen participants were salaried HCBC workers, five were professional nurses and the rest were non-professional, volunteer HCBC workers. The nurses had received a 2-year course in palliative nursing and had, in turn, had experience in informally training HCBC workers. The HCBC workers’ training in children’s palliative care was thus neither standardised nor accredited.

The photographs were used to elicit discussion and not as a source of factual information. During the individual discussions around the photographs, the opportunity was taken to engage each participant in a discussion around what they saw as practical ways of dealing with the challenges they faced in resource-poor settings. The researchers felt that the photographs were useful in overcoming language barriers and in dealing with emotionally challenging and sensitive issues. The two primary female researchers were involved in the entire research project from its inception which helped established a rapport with the HCBC workers, the majority of whom were female. The HCBC workers enjoyed the process and felt empowered to express their views and to become more aware of the needs and rights of the families they were caring for. Discussions with the participants were conducted in English, tape-recorded and transcribed to text verbatim. The transcripts were read and annotated for initial coding which were then collated to form thematic analysis. The team of researchers then used constant comparison to explore anticipated and emergent themes.

General results around the method pertained to its usefulness, enjoyment of the process, and the empowerment of the participants. Participants felt that the process allowed them to express themselves, increased awareness of issues such as patient’s health rights and facilitated decision-making processes. There were ethical challenges using the method which must be considered in future research. For example, the Human Sciences Research Council’s Research Ethics Committee reviewed all photographs and a decision was made not to use any photographs in which a person could be identified. The Ethics Committee felt that photographic subjects may not have a full realization of potential consequences of allowing photographs of themselves to be available in the public domain. This decision highlights an ongoing difficulty in using visual research methods in an area with high HIV prevalence. Home-based care workers also noted that families required a full explanation of why photographs were being taken and were concerned that the photographs may be sold for profit. Home-based care workers stated that they were generally able to explain the use of photographs and allay fears. None reported that a family had refused permission for photographs to be taken.

Discussion on the HCBC workers’ experience of caring for young children at home dealt with three major themes – the views of the HCBC workers on the home as a place of care, the advantages of palliative care, and the perceived challenges in home-care and palliative care. The HCBC workers noted a number of advantages of home-care over hospital or clinic-based care, including allowing the child to be with the family, to receive community support as well as access to traditional medicines. One participant took a photograph of her own daughter who was dying in a hospital and this facilitated discussion around the need for palliative care in a hospital setting as well as the need to provide support for carers. It also further emphasized the multiple roles of the HCBC worker, who in this case was the mother of a sick child herself in addition to serving the needs of those in her own community. The participant was grateful to have a copy of the photograph as a memento of her now deceased child. HCBC workers articulated many complex challenges in providing palliative care. They perceived that their ability to offer care was adversely affected by extreme poverty, inadequate housing and malnutrition, as well as fears of abandonment, isolation and stigmatization. A number of difficult emotions including feeling inadequate, upset, frustrated and threatened were also expressed - *“I took the photograph because I feel bad about these people living so hard, struggling but they can’t do nothing. No jobs, no nothing. I feel bad, very bad, very bad.”*

The method also highlighted gaps in training. For example, one participant did not recognize sores associated with HIV and was frightened to treat the sores. This participant also highlighted her sense of a lack of agency to facilitate change in her workplace: *“This child, she got the sickness from out of the country. I’m frightened, even to help her I’m frightened; but I do it because it’s my job and I can’t do nothing.”*

Other challenges included lack of medicines, concerns that caregivers sometimes neglect the care of their children, child abuse, physical dangers and concerns about the safety of some traditional medicines that caregivers were using.

### Formulating a model for the training and support package

Key messages were formulated to address the challenges identified. Storytelling was chosen as a culturally sensitive medium for delivering messages, a method that is accessible to a range of literacy levels. Stories are part of our cognitive repertoire for thinking, explaining, understanding and remembering; and for this reason listening and relating to stories is held to be one of the most important elements in learning [34–36]. Through reflection on and interpretation of narratives, experience can be constructed into pedagogical content so that storytelling can be used to inform and educate [37]. The benefits of stories for learning are well documented: facilitating learning in contexts with a high degree of uncertainty, conducive to problem solving [38, 39], more easily remembered than stand-alone abstract ideas [40], allowing people to understand things in meaningful and relevant ways [41], capturing tacit knowledge [42, 43], communicating complex ideas in simple and memorable ways [44], engaging both reason and emotion [45] and, finally providing the ability to communicate quickly, naturally and interactively [46]. We enlisted a ‘storyteller’, author and creative workshop facilitator who had worked with a number of local and international organizations to develop and facilitate creative training workshops in communities, particularly in the context of HIV and AIDS. In our training model, nine stories were developed to represent the nine key ‘take home’ messages that addressed the main challenges experienced by HCBC workers in supporting caregivers of sick young children (see Table 3). This model was presented in a series of four workshops with a total of 32 HCBC workers from four organizations. The 32 participants (who were previously involved in other stages of the project) included professional nurses, supervisors of HCBC workers, HCBC workers with a range of training, and volunteers. Six of the 32 participants were male. The model was also presented at two local and two international conferences where feedback from audiences was taken in account during the development of the package. Once a final draft was complete, the package was shared with experts in the field from two international universities for their comments and feedback.

**Table 3:** Challenges expressed by home- and community-based care workers and the key messages developed to address these challenges

Challenges	Key message
Caregivers often lose hope because they feel that their actions may be futile.	Giving a little means a lot: showing the caregiver that the things that she* does to care for the sick child are important, no matter how big or small
Caregivers are often stressed/burnt out.	Be kind to yourself: reminding the caregiver to look after herself so that she can better take care of the child
Many caregivers have to deal with poverty, geographical isolation, being unsure of what actions to take e.g. if you suspect that a child is being abused.	Ask for help: reminding caregivers that there are networks of support available to them and that they should seek help; mapping out networks of support with caregivers
Children often experience stigmatization, abandonment, discomfort and pain, neglect and abuse; caregivers may not know how to respond.	Listen to the child: helping caregivers understand how to listen to the child’s wants and needs, even if they are not expressed verbally
	Offer comfort to a distressed child: teach and support caregivers to give comfort to a sick child
Children may experience a range of common health problems that caregivers have to manage. These situations may be exacerbated by a lack of resources.	Prevent and treat: empowering and supporting the caregiver to be able to recognize the signs, then prevent and treat common problems that may arise
In many cases caregivers demonstrate an overreliance on the home- or community-based care worker. Professional boundaries may be blurred when this overreliance persists.	Empower: empowering the child as well as his/her caregivers and family members to find workable solutions to their problems
In many families children are not included in discussions about their health. Family members also express uncertainty about the future.	Prepare the child and the family: supporting the caregiver and the family to discuss the issue of sickness and death with a child in a manner that they are comfortable with, and preparing family members for what lies ahead
Often there are cultural clashes around issues of death and dying.	Remember: supporting the caregiver and family members to deal with the child’s death in culturally-appropriate ways and to remember the child

*The majority of home-based caregivers and primary caregivers who participated in this study were female. While we use the female pronoun throughout the text for ease of reading we recognise and encourage the contribution of males in home-based care and caregiving

## Results and discussion

### Description of the training and support package

The underlying principles of the package were to facilitate learning through practice in the home environment and to build partnerships between professional healthcare workers, HCBC workers, the wider community, families and other caregivers. The training and support package (see Table 4 for components of the package) contains practical guidance, techniques and tips and uses stories to facilitate the training of HCBC workers and to enhance the support of caregivers. The package is structured in four parts; each component of the package follows this structure but is tailored to suit the context in which it is used. During the development phase the course was delivered over 3 days which was adequate time to cover all material. Groups of between 10 and 15 participants were found to be manageable for both whole-group activities and smaller breakaway groups. The package^1^ includes material used to train trainers, material that these trainers use to then train HCBC workers, and material that the HCBC worker can use to guide their work in terms of supporting families/primary caregivers – and training primary caregivers to provide better care for the young sick child – and supporting HCBC workers to care for themselves.

**Table 4:** Components of the training and support package

Guide for Home-based Care Workers: a technical booklet that should be used by someone with background and training in palliative care to train home- and community-based care workers. Groups of HCBC workers should be trained on the content of the guide and how to deliver the course. These master trainers will, in turn, use the guide to develop their own training sessions as well as implementing its principles and methods in their own field work.
Training Manual for Home-based Care Workers: HCBC workers trained as master trainers use the training manual to facilitate the training of other groups of HCBC workers. The manual contains practical information about time frames, props or materials needed during each session, key learning outcomes, and activities that need to be followed to deliver the training. Vignettes and group activities have been designed to facilitate discussion, learning, and sharing.
Caregiver’s Toolkit: HCBC workers distribute toolkits to the families they support and use their own training manual to make the caregivers familiar with the toolkit. The toolkit is less technical and contains practical tips about how to keep germs away, how to breastfeed and prepare solid foods, advice on immunizations and growth monitoring for the child, when and what health check-ups the caregiver and child should receive, and dealing with common problems such as diarrhoea, fever and constipation. There are also activities for the HCBC and caregiver to work through together e.g. mapping circles of support.
Helpful Handouts: illustrated laminated handouts for caregivers containing important information on how to keep germs away, how and when to breastfeed and formula feed, food hygiene, how to recognize and deal with diarrhoea, fever, skin and mouth problems, and a number of danger signs that caregivers should watch out for that require the child to be taken to a health centre.
Information and Evaluation Sheets: managers and administrators are presented with information on the skills and competencies that HCBC workers should take away from the course and an evaluation sheet to assess the level of learning and retention achieved by those who have participated in the course.
Training and Support Certificates: master trainers, HCBC workers and caregivers receive certificates to demonstrate the competencies gained in the various knowledge and skill areas included in this course.

#### Part 1: Understanding palliative care in general and specifically, paediatric palliative care

This section deals with a number of foundational issues such as what is meant by palliative care, caregiver’s needs, children’s needs, and what HCBC workers and caregivers can do to meet the needs of young children.

#### Part 2: Supporting a caregiver to care for a child with HIV infection who is well

Information is provided on how HCBC workers can support caregivers to keep a child well, how caregivers can be supported to care for themselves, helping caregivers to decide when and how to disclose a child’s HIV status, and to show caregivers ways to ensure that the child continuously takes medication. Caregivers who have trouble accepting the HIV status of their children and report feeling depressed or under excessive emotional strain appear to have greater difficulties in complying with advice on childcare from counsellors [47]. Children may also have diminished capacity to meaningfully participate in their own healthcare [48]. The package promotes communication around illness and places emphasis on important contextual factors, such as the age and development status of the child, and family and cultural understanding of illness and death, an area that requires much work [8].

In Part 2 issues such as nutrition and feeding – including breastfeeding – are covered to ensure that the child gets optimal nourishment [6, 47]. Over and above the physical needs of a sick child, it is important to nourish their emotional well-being. Close and regular interaction with a loving and responsive caregiver is crucial and it is important to encourage caregivers and children to play together [6].

#### Part 3: Supporting a caregiver to care for a child with HIV infection who becomes sick and gets better

Research shows that children with HIV infection often experience a wide array of symptoms and on any given day HCBC workers can face a range of physical problems that cause distress in young children and for their caregivers [49]. The section focuses on how to treat common problems, including pain, crying and distress, fever, dry mouth, nausea and vomiting, constipation, rash or itchy skin, and coughing, as well as information on when to refer a sick child to a clinic, how to deal with the special problems that a child on ART may face, how to prevent child abuse and neglect, and how to manage reported cases of child abuse and neglect [50]. Part 3 uses the ‘assess, advise, assist, and arrange’ technique. For each problem, HCBC workers are encouraged to assess the situation by looking at, listening to and feeling the child; to advise the caregiver on what they can do to help the child; to assist and finally, to arrange to either have the child referred to a clinic or to set up another visit to the home. The technique was adapted from the 5A’s of Behavioural Change [51]. The first step of approach is ‘ask’ which was eliminated in this instance once HCBC workers advised that they generally identify a problem through a number of verbal and non-verbal methods when dealing one-on-one with young children. A technique like this, with a concrete set of sequential steps to apply in different circumstances, is useful in the HCBC context where care workers face a wide range of challenges with few resources.

#### Part 4: Supporting a caregiver whose child with HIV infection becomes sick and may not get better

Part 4 is based on supporting the caregiver of the young child with HIV who may not get better. Importantly, this section begins with reasons why a caregiver may want to keep a sick child at home, as opposed to intermittent care in a hospital. It is here, in Part 4, that the nine messages each delivered through a story are used to support the caregiver and the sick child. This section begins by encouraging the caregiver to acknowledge the many things they are already doing to care for the young child which are of great consequence; this is important for empowering caregivers and relieving a sense of futility they may experience. A study in Uganda found that caregivers often feel helpless when their children go through periods of illness, fearing that their child may die at any moment [47].

Part 4 goes on to cover pain management, soothing distress and providing comfort, recognizing the signs of death, answering difficult questions and helping the family to prepare both practically and emotionally for the death of a child. Included are tools for pain assessment, a list of health check-ups and how often they should be performed, as well as a basic list of medicines that can be used in the care of sick, young children at home. Chronic and often severe pain is commonly seen in children with HIV infection, particularly when CD4 counts are low [6]. However, the use of analgesics is still limited in some parts of sub-Saharan Africa due to suspicions that pain medicine may be addictive or dangerous for children [52]. Considering that pain is one of the most frequently experienced symptoms presented by children with HIV infection [53], it is important to emphasize accurate assessment and effective management of pain.

The training and support package, while being culturally sensitive, includes some spiritual aspects. For example, training sessions can be opened and closed both in prayer and in song. Tending to the spiritual needs of HCBC workers, families, caregivers, and the child is usually overlooked, although it is often placed high on the list of needs reported by individuals involved in the care of sick young children [54]. Care workers are encouraged to respect the religious and cultural beliefs of the families they care for, especially in discussing death and dying and the family’s wishes after the child’s death. Families may want to involve traditional healers in the care of their child. The package advocates for this involvement when it is safe and does not contradict medical care. In Botswana, interviews with a number of primary caregivers revealed that traditional medicine played an important role in their care of terminally or chronically ill family members [54].

The nine key messages considered essential to improving the quality of care which young children receive have been integrated into a complete training and support package which follows this four part structure and consists of the components described in Table 4.

### Evaluation of the use of the training and support package

An independent evaluator, fluent in Zulu and with extensive experience in the field, was commissioned to conduct an assessment of the use of the training and support package in the homes of families caring for sick young children. The evaluator was made familiar with the training and support package over a number of weeks and presented with i) a checklist to be used during observational visits with the HCBC worker of what should be visible in the interaction between HCBC workers and the family if the package was successful in producing the desired results, and ii) a semi-structured questionnaire designed to evaluate the views of the HCBC workers on their use of the training and support package subsequent to its use.

Two organizations were chosen randomly and approached to participate. Thirteen of their HCBC workers agreed to participate in the evaluation. All 13 HCBC workers had prior training in home-based care and were female. The youngest care worker was 21 and the oldest was 55 years old, with a median age of 42. The HCBC workers were trained using the training and support package. After training, home-based care workers were requested to use this package to provide support for at least two caregivers of young children with HIV infection at home over a period of 2 months. This was over and above their usual workload and HCBC workers were not given any additional financial compensation. The evaluator accompanied the care workers on two home visits per carer and interacted with the primary caregivers receiving the support, eliciting their views on the package. The evaluation focused on the HCBC workers experience of delivering the package to the primary caregiver and the primary caregivers’ experience of this delivery. After the 2 months, the HCBC workers were interviewed by the evaluator at their place of work using a semi-structured questionnaire. Twenty primary caregivers completed the full process – two visits and an interview (solely with the caregiver and evaluator). The majority of caregivers (16) were maternal grandmothers over the age of 65; two caregivers were young women under the age of 25, one caregiver was a father and the remaining caregiver was a grandfather. Sixteen of the children’s mothers had died and the fathers’ whereabouts were either unknown or they were not active in the child’s life. Thirteen of the children being cared for were already on antiretroviral therapy.

Using the checklist, the evaluator’s observation also focused on the interaction between the primary caregiver and the child – focusing on aspects of the training to be implemented in the primary caregiver’s provision of care. Of particular importance was the retention of messages and principles espoused in the training and support package and perceptions of the usefulness of the course and the package. Findings reflected that both HCBC workers and caregivers felt that the course was useful in delivering the key messages around palliative care at home for young children.

The evaluator compiled two reports from her field notes which were submitted to the research team. The questionnaires on the views of the HCBC workers and caregivers were captured electronically and synthesized.

#### HCBC workers’ views on the training and support package

There was a general desire to pass on the principles and messages that the training and support package espoused:

*“The trainer really took her time to ensure we understood all. I learnt a lot. I realised that there’s a lot different between caring for a child and caring for a sick child. I’m so excited to teach and share the information I got.”*

The course appeared to give HCBC workers a structured way of teaching and supporting caregivers in caring for their young children at home. Most HCBC workers appreciated the participatory nature of the package and they felt respected and that their experiences were valued. The care workers were also able to use the package in their own lives:

*“I learnt a lot of new things I didn’t know; I learnt how to deal with children in my own family before I go out and apply these learnings/messages outside”*

*“In life it’s the little things that we don’t give much notice to, like listening to a child, comforting a child. For me the course was so useful because it helped me allow myself to think about and remember my own mother who I lost when I was very young. No one told me it was ok to think about her and therefore I deprived myself. I think because I didn’t know how they’ll deal with me if I started asking questions and now I know exactly how to handle children and their questions.”*

One criticism was that the course was too short to allow trainees to fully grapple with all of the material. However, all participants agreed that they had received a good foundation for caring for sick young children, including those suffering from AIDS, at home. Extending the training to primary caregivers highlighted that training should always be pitched at the level and pace of the recipient. Since most of the caregivers were older, the care workers felt that together they needed more time to engage with the material at the pace of the caregiver. In addition to differences in learning needs, older caregivers can often experience depression, exhaustion and neglect of their own health due to the magnitude and multiplicity of duties they have to perform [54]. Working through the materials which were in English also slowed delivery of the key messages and principles and numerous requests were made to have the package translated into Zulu. Based on this feedback the research team recommends that the period of training be adjusted according to the trainer’s discretion based on the language, literacy and other needs of participants.

Male HCBC workers often report experiencing unique problems around caregiving. As men in the community, their intentions for caring for sick children were questioned; they were regarded with suspicions of seeking vulnerable children to exploit, being gay, or wanting to take the wife of the family that they provided care for. The developers of the package sought to address these concerns by emphasizing that caregiving is both the work of men and women. One home-based care worker remarked:

*“I learnt that a man can take care of a child. I used to be very scared of men caring for children but I learnt it was okay since in other families primary caregivers are men.”*

#### Caregivers’ views on the training and support package

*“At first I was terrified when she (the home-based care worker) introduced this course to me. I thought why does she want me to learn about very sick children? What did she see in my child that I didn’t? Does she think that my child is going to die? These changed though as I started seeing the value of the course that it is not only about when my child is very sick but I could even prevent some of the problems from arising.”*

Caregivers appreciated that the course did not focus solely on sick and dying children but also dealt with the well child, and more importantly, the caregiver:

*“The course helped me learn the importance of loving myself first and caring for myself before I can love and care for others. It taught me to talk to people, children and that if I am angry I should not take it out on my child and learn to talk about things that have happened in our family.”*

Caregivers believed that the package went a long way to helping them develop strategies of interacting with children, especially sick children, and they felt that going through the training improved their relationship with their children. Consistently, caregivers highlighted the ‘*listen to the child’* message as being very significant to them.

*“The course taught me a lot about a child who is sick at home and how to care for them. At the hospital a child is not free to talk or ask for anything, home is better. It helped me know how to help even if a child cannot talk for themselves, to help even the neighbour’s child if I see the need.”*

The caregivers felt that the stories included in the material were useful and culturally appropriate. Many of the older caregivers appreciated the method as a way of preserving the culture of “talking around the fire” and felt that storytelling was a much easier way of passing on learning to others and educating and entertaining children.

## Conclusion

Children are usually hospitalised during serious illness which requires palliation, but hospitalization may present problems for both the child and the family. The child is away from loved ones and is in unfamiliar surroundings at a time of great vulnerability. The family may have to spend scarce resources to visit and may not be able to spend quality time with their child. The child may be discharged when further hospital treatment is unlikely to lead to recovery. Looking after a very sick child at home is enormously difficult for families, especially if they have limited infrastructure, little access to services and very few resources. Although there may be community workers who could help, few of them are equipped or trained to give advice regarding palliation. This home-based paediatric palliative care training and support package is designed to help HCBC workers who are in a position to support families in provision of quality palliative care. The comprehensive training and support package is presented within an adult-friendly learning framework that is accessible to a range of literacy levels. The underlying principle is to facilitate learning through practice in the home environment and to build partnerships between professional health care workers, HCBC workers, families, sick children and caregivers. The training guide contains practical guidance, techniques and tips. One of the main strengths of the package is it can be used in its entirety or the individual components can be used separately as resources and need dictates. A unique element is the use of stories to facilitate the training; there is a story for each important message to make it easier to understand and remember. The stories could be replaced by other locally relevant stories with the same message and although the package is most valuable as a whole, parts of it can be useful separately. An initial evaluation of the training and support package was positive; showing support for both the content and the structure of the package, as well as the inclusion of stories to help deliver key messages crucial to the provision of palliative care. Since the package was launched there have been widespread calls for its wider dissemination.

### Ethics and consent to participate

Ethical clearance was obtained from the HSRC Research Ethics Committee (REC 2/19/09) and written consent was obtained from all the organizations and families involved in the research project.

### Consent to publish

Not applicable.

### Availability of data and materials

Data and information gathered during the process of developing the training and support package, as well as electronic copies of the training and support package, are available on request from the corresponding author.

## Additional file

Additional file 1: The Nine Messages. (PDF 799 kb)

## Abbreviations

HCBC: Home- and community-based care

## Footnotes

1 The corresponding author can be contacted for access to the full training and support package.
