# Supplementary material for: Development and pilot evaluation of a home-based palliative care training and support package for young children in southern Africa
Source: BMC Palliat Care. 2016 Apr 9;15:41. doi: 10.1186/s12904-016-0114-7 (PMC4826506; doi:10.1186/s12904-016-0114-7)
Supplement: Additional file 1: — The Nine Messages. (PDF 799 kb) [file 12904_2016_114_MOESM1_ESM.pdf]

1. *Giving a little can mean a lot*
2. *Be kind to yourself*
3. *Ask for help*
4. *Listen to the child*
5. *Offer comfort to a distressed child*
6. *Prepare the child and the family*
7. *Prevent and treat*
8. *Empower*
9. *Remember*

Message 1 - Giving a little can mean a lot

The home-based care worker will tell you a story called *"Daisy Flowers"* and you will discuss this story.

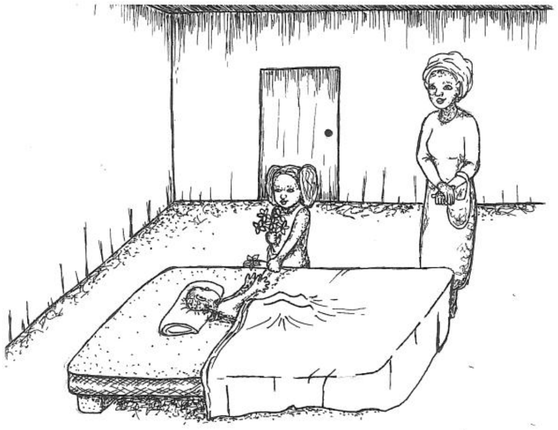

Giving a little means a lot

Draw a picture of a time when you did something small and it made a big difference to someone's life.

Don't give up hope. Draw one thing that you can do today if you are feeling sad and lonely.

Message 2 - Be kind to yourself

Your home-based care worker will tell you a story called *"The Big Flowerpot"* and you will discuss this story.

Be kind to yourself

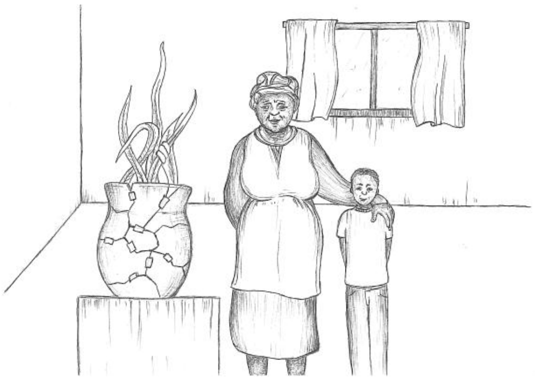

Know that you cannot be perfect. Allow others to help you.
Know that things do not always turn out the way you expect them to.

We will look at the following:

- *What happens if you are not kind to yourself?*
- *How can you be kind to yourself?*

What happens if you are not kind to yourself?

If you are not kind to yourself you may become ill in your body, your mind and in your emotions.

Write down what happens to your body, mind and emotions when you are stressed?

**What happens to my body?**	
**What happens to my mind?**	
**What happens to my emotions?**	

How can you be kind to yourself?

Draw three things that you can do to be kind to yourself, to rest and to look after yourself.

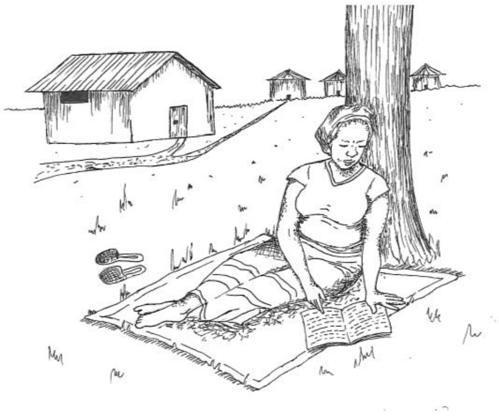

Message 3 - Ask for help

Your home-based care worker will tell you a story called *"The Empty Cup"* and you will discuss this story.

Ask for help

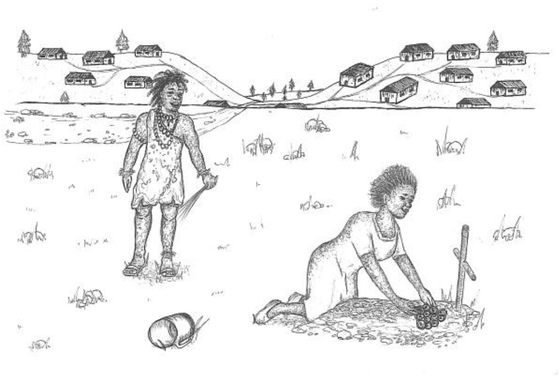

We will look at who you can ask for help.

Circles of Support

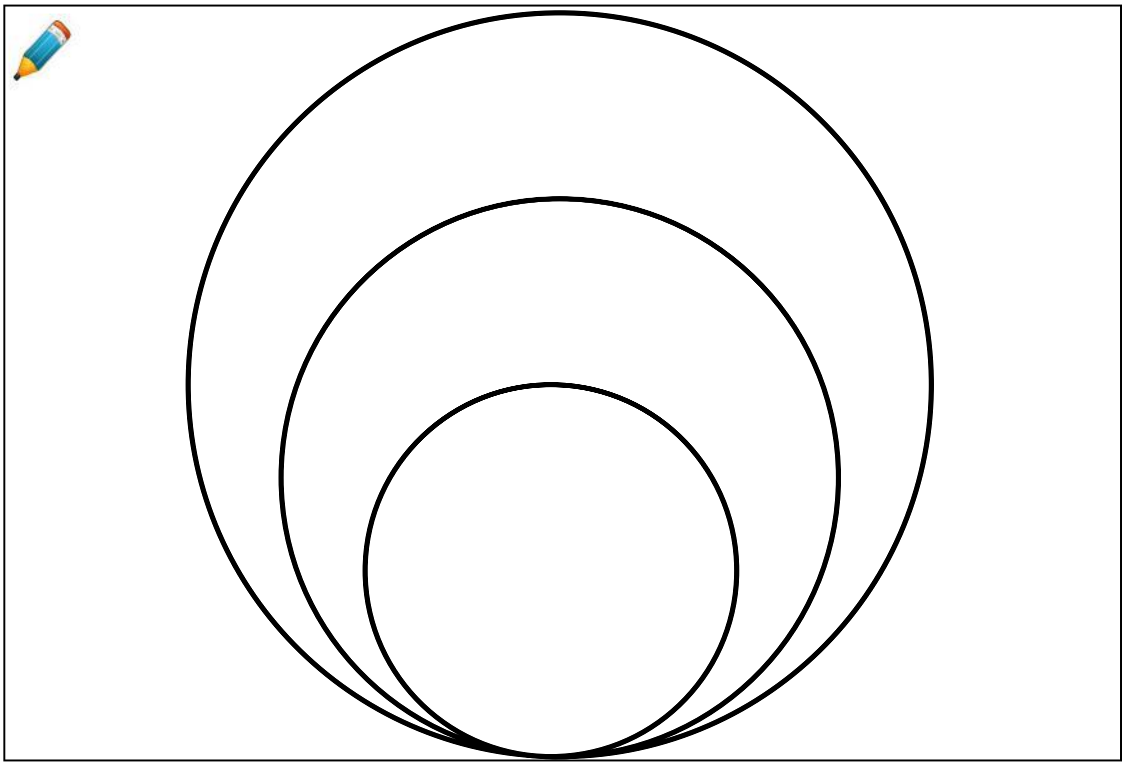

Draw yourself and a sick, young child in the small centre circle.

Draw your family and friends in the next circle.

Draw other people who could help you in the biggest circle.

Together you and the home-based care worker will draw a map of your area and together you can look at where help is available.

A Community Map

It is important that you know who to ask for help.

Message 4 - Listen to the child

Your home-based care worker will tell you a story called *"The Snake's Ears"* and you will discuss this story.

Listen to the child

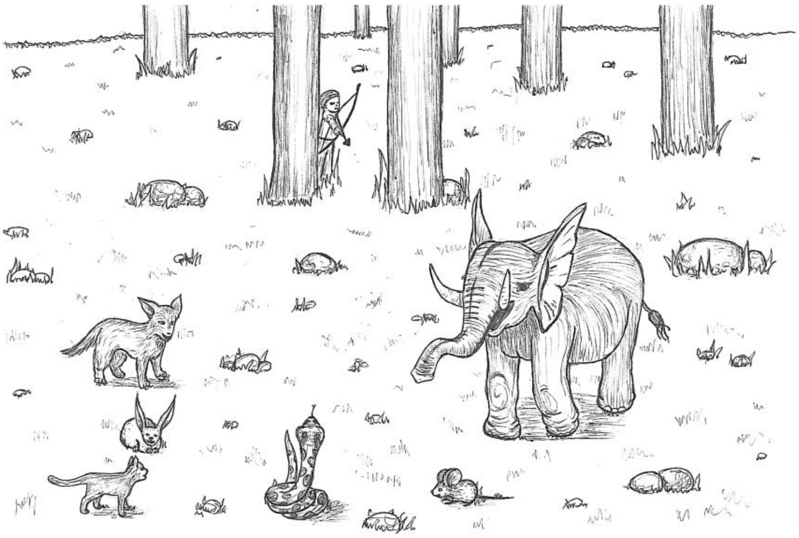

Do not listen to your sick child with only your ears, but with your heart and understanding too.

Watch the child carefully.

Listen to children with the ears on your head and the ears in your heart.

We look at the following:

- *Why is it good to listen to a young child?*
- *How can you listen well to your young, sick child?*

Why is it good to listen to a young child?

Draw three reasons why it is good to listen to a sick, young child.

How can you listen well to your young, sick child?

Draw five ways that you can listen well to a sick, young child.

Message 5 - Offer comfort to a distressed child

Your home-based care worker will tell you a story called "*Snow White Flower*" and you will discuss this story.

Offer comfort to a distressed child

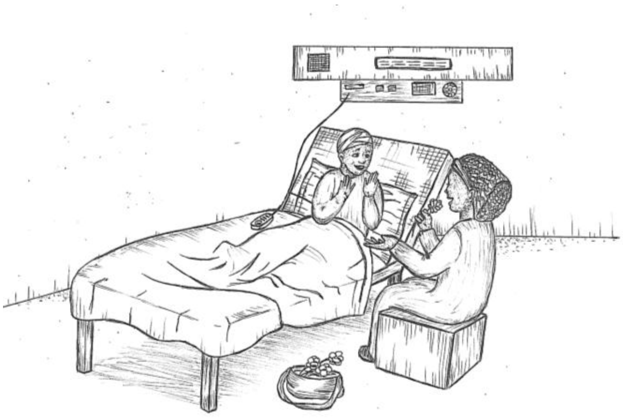

You can always offer some comfort to your distressed child.

Draw three things that you could do with a distressed young child to comfort them.

Message 6 – Prepare the child and the family

Your home-based care worker will tell you a story called "*Flowers from Heaven*" and you will discuss this story.

Prepare the child and the family

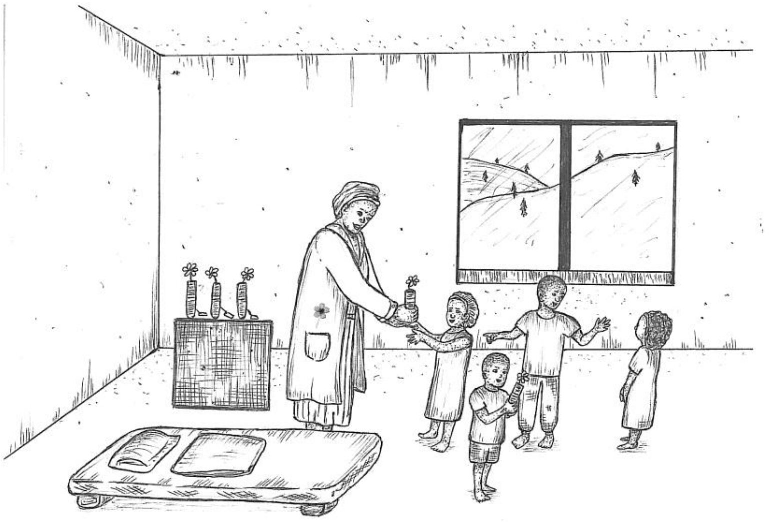

Your sick child may ask you about illness, dying or death.

You may want to tell other children in your family about illness, dying and death.

We will look at the following:

- *Why it might be good to tell children about illness, dying and death*
- *How to talk to young children about illness, dying and death*
- *What you can do if a young child does not want to eat or drink*
- *What happens immediately before death and after death*

Why might it be a good thing to talk to a young, sick child about illness, dying and death?

Draw three reasons why it may be good to tell a sick, young child about illness, dying and death.

Why may it be a good thing to talk to your sick child's brothers, sisters and friends about illness, dying and death?

Draw three reasons why it might be good to talk with brothers, sisters and friends about illness, death and dying?

How to talk to young children about illness, dying and death

Instead of talking about dying you could tell your child a story about something that goes away and does not return.

We find the story below helps us to talk to sick young children and their brothers and sisters about illness, dying and death. You can get all the children together to share the story.

John and James

John had a special friend and his name was James. They played together at school. Sometimes John got sick and could not go to school. Sometimes he felt angry and sad because he had to stay in bed. Sometimes he felt scared because he knew he had a germ in his blood.

Ask one child to draw a picture of John.

John had to go to hospital and the doctors and the nurses at the hospital were kind. John missed his friend James.

Ask another child to draw a picture of John at hospital.

One day the doctor told John that he would not get better. John was very scared. He cried a lot and he told James all about what the doctor had said. John's granny told them a story called water-insects and dragonflies.

Water-insects are tiny creatures that live under the water. Sometimes the water-insects watch as one of their friends climbs up a large plant and disappears above the surface of the water. The friend does not return to them and they never see their friend again. The water-insects wonder what happened to their friend.

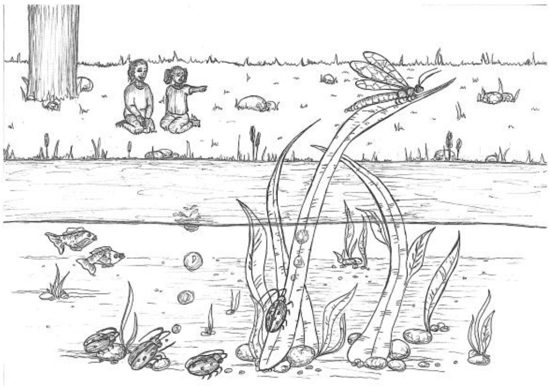

People that live on the land look at the water-insect as it climbs up the large plant and out of the water. They see it sit in the bright sunlight. They see it slowly change. It develops shining wings and becomes a beautiful dragonfly. It flies off happily to meet other dragonflies. Sometimes it remembers its friends below the surface but it cannot go back to them.

James said to John "I understand that the dragonfly cannot return to visit his friends. I will never forget you." Together they drew a picture of themselves.

Ask one child to draw a picture of John and James.

A few months later John died. His brothers and sisters and his friend, James, were very sad. They cried for a long time. James looked at the picture that he and John had drawn. He smiled and said, “I love you John”.

Ask one child to draw a picture of James.

Your home-based care worker will support you if you wish to discuss illness, dying and death with your young children.

What can you do if a very sick child does not want to drink or eat?

A very sick child, who is close to dying, does not need much fluid or food.

Your caregiver will tell you about the Aloe Flower.

Sometimes nature tells us not to give too much fluid or food.

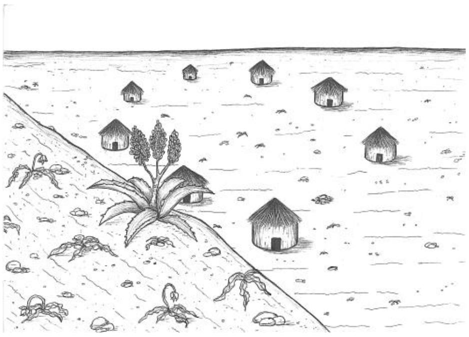

What happens immediately before death and after death?

These things may be difficult to talk about. If you would like to talk about them please tell your home-based care worker and she will cover these topics with you.

If you don’t want to talk about this, don’t worry. Your home-based care worker will respect what you want to do.

Message 7 - Prevent and treat

Your home-based care worker will tell you a story called *"The Red Moon"* and you will discuss this story.

Prevent and treat

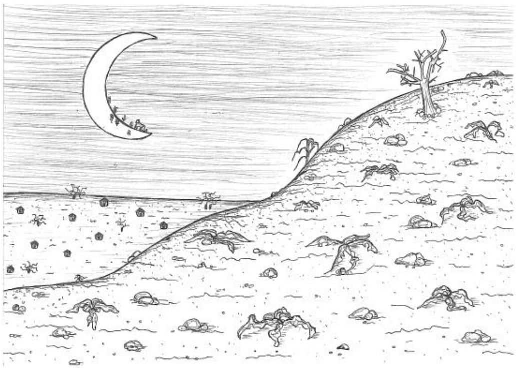

It is better to prevent problems before they arise. Then, treat problems that do occur.

We will look at the following:

- *Pain in a young, sick child*
- *Preventing and treating pressure sores*
- *Preventing stiff joints*

Looking at pain in a sick, young child

If your child is more than 4 years old you can use these pictures. Ask your child to point to which picture best shows their pain.

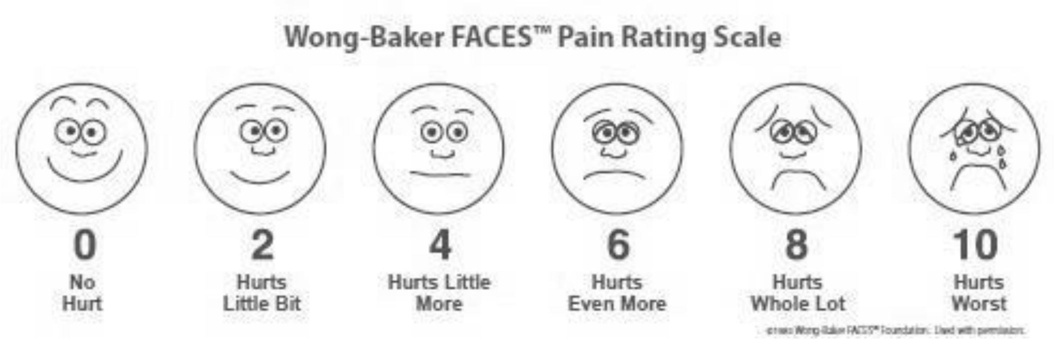

Face 0	Is a happy face because your child has no pain.
Face 2 to Face 4	Your child has pain that hurts just a little bit.
Face 6 to Face 8	Your child has pain that is upsetting.
Face 10	Your child has very severe pain that is very upsetting.

Together you and your home-based care worker will look at what is causing the pain and what to do about the pain.

If your child is younger than 4 years, you and the home-based care worker need to watch the child carefully and see if there is pain or not.

Here is what you must watch for and the home-based care worker will help you with this.

Looking for Pain in a Young Child

**What to look at:**	**No pain**	**A little pain**	**Lots of pain**
Look at the face			
Look at the legs			
Look at how the child lies			
Listen to the child's cry			
Can the child be comforted			

Preventing and treating pressure sores

Together you and the home-based care worker will also look at preventing and treating pressure sores.

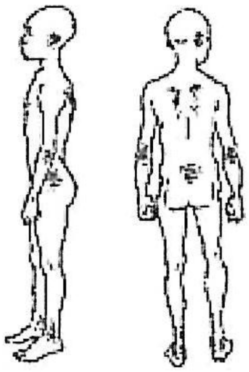

Preventing stiff joints

The home-based care worker will also discuss how you can deal with stiffness.

Draw a picture of how to prevent stiff joints.

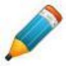

Message 8 - Empower

Your home-based care worker will tell you a story called *"The Boy and the Flowers"* and you will discuss this story.

Empower

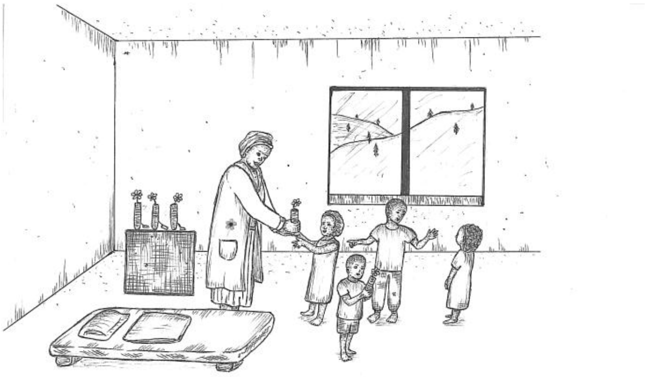

Even your sick child can come up with a possible solution to problems. Work out how you can make the solution happen. Earlier we looked at your child's main problem and your main problem.

Draw one thing you can do about your child's problem.

Draw one thing you can do about your own problem.

Message 9 - Remember

Your home-based care worker will tell you a story called *"The Wax Child"* and you will discuss this story.

Remember

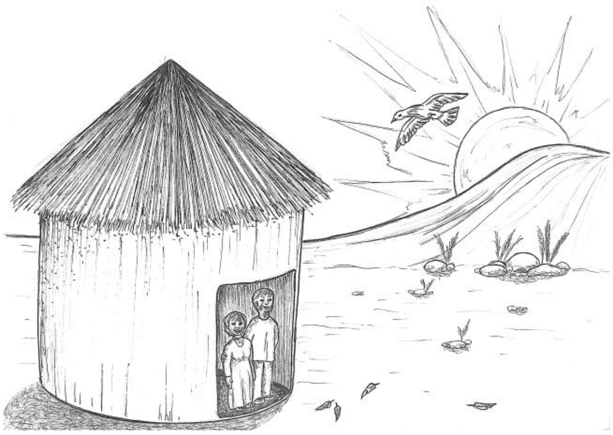

You can find much comfort in remembering those who died.

You can prepare for the death of a loved one.

We will look at the following:

- *A memory box*
- *You grieving the loss of your young child*
- *Other children grieving*

A memory box

Your home-based care worker will discuss the idea of a memory box with you.

Draw a picture of what your memory box would look like.

You grieving the loss of your young child

Draw a picture of how you may feel if a young child dies.

Other children grieving

Draw a picture of how young children at home may feel if someone they know dies.

We may need to ask a nurse or doctor for help if we notice any of the following in a young grieving child:

- The older child begins to use baby talk, sucks their thumb, and has incontinence of urine
- Has severe nightmares
- Harms himself or others
- Becomes very quiet
- Will not eat
- Cries most of the time
